# Isolation and Characterization of Immune Cells from Mouse Skin in Steady State and During Inflammation

**DOI:** 10.1002/cpz1.70516

**Published:** 2026-10-31

**Authors:** Darawan Tabtim‐On, Mathieu Vetter, Elodie Segura

**Affiliations:** ^1^ Institut Curie, PSL Research University, INSERM, U932 Paris France; ^2^ Institut Necker Enfants Malades Université Paris Cité, INSERM, U1151 Paris France

**Keywords:** immune cells, mouse, skin, wound

## Abstract

Immune cells in the skin play an important role in maintaining tissue homeostasis and responding to tissue stress and injury. However, isolating immune cells from skin can be challenging because of its dense extracellular matrix and the potential effects of prolonged enzymatic digestion on cell viability and surface markers. Here, we describe a combined mechanical and enzymatic method for isolating immune cells from mouse skin for downstream flow cytometry and RNA sequencing applications, together with a flow cytometry gating strategy for the identification and characterization of skin myeloid cell populations. The long digestion uses an overnight dispase II treatment followed by collagenase IV and DNase I digestion to facilitate epidermal and dermal separation and subsequent tissue dissociation, while the fast‐digest bypasses the overnight step and uses Liberase TL to obtain cells within approximately 2 hr. The fast‐digest workflow reduces processing time and provides an alternative approach for applications requiring RNA‐based analyses. Together, these workflows provide flexible methods for studying immune cell composition and transcriptional responses during steady‐state and injury. © 2026 The Author(s). *Current Protocols* published by Wiley Periodicals LLC.

**Basic Protocol**: Isolation of skin immune cells for flow cytometry

**Alternative Protocol**: Isolating skin immune cells using a “fast‐digest” technique

## Introduction

The skin is a complex tissue containing diverse immune cell populations that respond to changes in the tissue environment, including mechanical stress and injury (Kabashima et al., 2019; Peña and Martin, 2024). Studying these responses requires efficiently isolating immune cells while maintaining cell viability and surface marker expression for downstream analysis. However, the dense extracellular matrix of the skin makes immune cell isolation challenging. Furthermore, prolonged enzymatic digestion can affect cell‐surface marker expression and transcriptional profiles (van den Brink et al., 2017).

Here, we describe a combined mechanical and enzymatic isolation method optimized for mouse back skin, but also applicable to ear and footpad skin. The protocols detail two workflows adapted to different downstream applications: a long digestion for comprehensive immune cell profiling by flow cytometry and a short digestion (“fast digest”) designed to minimize processing time for bulk or single‐cell RNA sequencing. The long digestion uses an overnight dispase II treatment followed by collagenase IV and DNase I digestion to maximize tissue dissociation and immune cell recovery, whereas the fast digest bypasses the overnight step and uses Liberase TL to obtain cells within ∼2 hr.

We routinely apply these protocols to study mild skin stress, such as mechanical stretching (Benaouda et al., 2025), and severe injury, such as full‐thickness excisional wounding. Together, these workflows provide complementary approaches for studying immune cell composition and transcriptional responses in steady‐state and injury conditions, depending on the downstream application.

*NOTE*: Keep the collected skin samples cold during collection and transport to minimize changes in cell viability and activation state.

*NOTE*: Process samples as consistently as possible, including keeping the time between skin collection, enzymatic digestion, and flow cytometry staining as uniform as possible.

*NOTE*: Ensure that the skin is fully immersed in the digestion mix during the incubation.

*NOTE*: All protocols involving animals must be reviewed and approved by the appropriate Animal Care and Use Committee and must follow regulations for the care and use of laboratory animals. Appropriate informed consent is necessary for obtaining and use of human study material.

## ISOLATION OF SKIN IMMUNE CELLS FOR FLOW CYTOMETRY

This protocol describes the isolation of immune cells from mouse skin for downstream flow cytometry analysis. The method involves an overnight dispase II treatment to facilitate separation of the epidermis and dermis, followed by mechanical disruption and enzymatic digestion with collagenase IV and DNase I to obtain a single‐cell suspension. The protocol is optimized for mouse back skin but can be adapted to other skin sites. The isolated cells can be used to characterize and quantify skin immune populations, including resident and infiltrating myeloid populations, by flow cytometry.

### Materials

Mice from which to obtain skin samples (we use C57BL/6J mice, typically aged 6‐12 weeks)
1× phosphate‐buffered saline (PBS), ice cold
Skin digestion medium (see recipe)
100 mg/ml dispase II (Roche, 04942078001)
10 mg/ml DNase I (Roche, 11284932001)
20 mg/ml collagenase IV (Sigma‐Aldrich, C4‐28‐100MG)
FACS buffer (see recipe)
LIVE/DEAD Fixable Blue Dead Cell Stain Kit (Invitrogen, L23105)
OneComp eBeads Compensation Beads (Invitrogen, 15242448)
Antibodies relevant to your experimental question, at appropriate dilutions (Table 1)

Flat‐tip forceps
Surgical scissors
12‐well tissue culture plates
Ice and ice bucket
Kimwipes
Weighing container
Laboratory balance reading in 1‐mg increments (suitable for weighing skin samples typically massing ∼0.1‐0.3 g)
Refrigerator, 4°C
Incubator, 37°C
Yellow tube rack
50‐ml Falcon tubes
40‐µm‐pore‐size cell strainers
5‐ml glass pipets
Centrifuge
96‐well plates with conical wells
Flow cytometer
Compensation beads
FlowJo software

**Table 1:** Antibodies for Flow Cytometric Detection of Myeloid Cell Populations

Antibodies	Manufacturer	Cat. no.	Clone
BV421 rat anti‐mouse CD11b	BioLegend	101235	M1/70
BV605 rat anti‐mouse Ly6G	BioLegend	127639	1A8
BV785 rat anti‐mouse I‐A/I‐E (MHC class II)	BioLegend	107645	M5/114.15.2
FITC rat anti‐mouse CD45	BioLegend	103107	30‐F11
PerCP/cyanine5.5 rat anti‐mouse F4/80	BioLegend	123128	BM8
PE rat anti‐mouse CD26	BioLegend	137804	H194‐112
PE/cyanine7 rat anti‐mouse/human CD64	BioLegend	139314	X54‐5/7.1
Alexa Fluor647 rat anti‐mouse Tim‐4	BioLegend	130008	RMT4‐54
Alexa Fluor700 rat anti‐mouse Ly‐6C	BioLegend	128023	HK1.4
APC/Fire750 rat anti‐mouse CD326 (Ep‐CAM)	BioLegend	118229	G8.8

#### Day 1: Obtaining skin biopsies (timing: ∼2 min per mouse)

For experiments involving skin wounding, the skin area of interest should be shaved and depilated before performing the wound procedure. Collect the skin samples as described below after the experimental wounding procedure.

1 Euthanize the mice by cervical dislocation.
2 Using flat‐tip forceps, gently pinch the back skin and excise the area of interest using scissors. Immediately place the skin samples into a 12‐well plate containing 1 ml ice‐cold 1× PBS. Keep the plate on ice during transport.
3 Collect an additional skin sample to be used for single‐stained viability controls during flow cytometry.

#### Day 1: Enzymatic dissociation of the skin (timing: overnight, i.e., 12‐16 hr)

In this stage of the protocol, the entire piece of skin is incubated in dispase II digestion solution overnight to facilitate separation of the epidermis and dermis. This step improves subsequent tissue dissociation and allows better recovery of Langerhans cells and other resident immune cells located in the epidermis. Complete enzymatic digestion is performed the following day.

*NOTE*: Unless otherwise indicated, these steps are conducted at room temperature in an open laboratory space.

4 Prepare skin digestion mix I (see recipe in Reagents and Solutions) by adding dispase II at a 1/50 dilution of the prepared stock solution to skin digestion medium.
5 Transfer 1 ml of the digestion mix into each well of a 12‐well plate.
6 Weigh each skin sample by first placing a clean weighing container on the scale and taring the balance. Gently tap the skin with a clean Kimwipe to remove excess PBS, and then weigh the skin.
  The tissue weight is used to normalize immune cell counts to the skin mass and report the results as cells per gram of skin. Depending on the size of the area of interest, ∼0.1‐0.3 g of skin is typically processed per well.
7 Immediately transfer the skin sample into the prepared dispase II digestion mix with the dermal side down.
8 Incubate the plate at 4°C overnight (12‐16 hr), leaving it stationary in the refrigerator or cold room.

#### Day 2: Mechanical and enzymatic digestion of the skin (timing: ∼2 hr)

In this stage, the skin is mechanically disrupted and enzymatically digested to obtain a single‐cell suspension for flow cytometry.

*NOTE*: Unless otherwise indicated, these steps are conducted at room temperature in an open laboratory space. Turn on the incubator at 37°C beforehand to ensure that it has reached and maintained the required temperature.

9 Prepare skin digestion mix II (see recipe in Reagents and Solutions) by adding DNase I at a 1/20 dilution and collagenase IV at a 1/13 dilution of the prepared stock solutions to skin digestion medium. Keep the digestion mix on ice until use.
10 Transfer the skin into a 12‐well plate and use surgical scissors to finely cut up the skin directly in the well. We usually cut the skin ∼150‐180 times; smaller skin pieces require fewer cuts.
11 Add 1 ml of skin digestion mix II (from step 9) to the cut skin and gently swirl the plate to mix.
12 Close the plate lid and incubate at 37°C for 30 min.
13 Add another 1 ml of skin digestion mix II to each sample and gently swirl the plate to mix.
14 Close the lid and incubate at 37°C for another 30 min.
15 During the incubation, prepare one 50‐ml Falcon tube and one 40‐µm‐pore‐size cell strainer for each sample on a yellow tube rack.
16 Once the incubation is complete, transfer the partially digested skin mixture using a 5‐ml glass pipet through a 40‐µm cell strainer into the corresponding 50‐ml Falcon tube.
  The wide opening of the glass pipet facilitates aspiration and transfer of the partially digested tissue.
17 Centrifuge for 5 min at 450 × *g*, 4°C.
18 Carefully discard the supernatant and resuspend the pellet in 200 µl of FACS buffer.
19 Transfer the cell suspension to the wells of a 96‐well plate with conical wells for further staining.
20 Centrifuge plate for 5 min at 450 × *g*, 4°C, and then proceed immediately to the next step.
  After this step, the cell pellets are ready for live/dead staining or conventional antibody staining for downstream flow cytometry. We are mainly interested in myeloid cell populations in the skin; therefore, our antibody cocktails consist mainly of antibodies used to detect these populations.

#### Day 2: Flow cytometry staining of isolated immune cells from the skin (timing: ∼1.5 hr)

In this stage, the isolated cells are stained with a viability dye and antibodies for flow cytometry analysis.

*NOTE*: Keep the cells on ice during staining steps unless otherwise indicated.

21 Stain the cells with 1/1000 Live/Dead Fixable Blue stain prepared in PBS, in a 100‐µl volume.
22 Incubate on ice for 10 min.
23 Wash the cells with 100 µl FACS buffer per well, and pipet up and down once or twice to resuspend the cells.
24 Centrifuge for 5 min at 450 × *g*, 4°C.
25 Meanwhile, prepare appropriate antibody cocktails for your experimental question (see Table 1).
26 Carefully remove the supernatant from each well.
27 Stain the cell pellets with 50 µl of antibody cocktail.
28 Incubate on ice for 30 min.
29 Wash the cells with 100 µl FACS buffer and pipet up and down once or twice.
30 Centrifuge for 5 min at 450 × *g*, 4°C.
31 Carefully remove the supernatant from each well.
32 Resuspend the contents each well in 100 µl FACS buffer. The cells are now ready for flow cytometry acquisition.
33 Prepare single‐stained controls using compensation beads or cell samples if preferred.
34 Acquire the samples using a flow cytometer. Before acquiring the samples, set the appropriate voltages and verify that the controls are correctly detected.
35 Analyze the data using FlowJo software.

## ISOLATING SKIN IMMUNE CELLS USING A “FAST‐DIGEST” TECHNIQUE

To minimize the time cells are exposed to tissue processing conditions, we developed a “fast digest” approach that bypasses the overnight dispase II incubation used in the basic protocol. Instead, this approach combines mechanical disruption with an additional enzymatic digestion using Liberase TL, along with collagenase IV and DNase I, to obtain a single‐cell suspension within ∼2 hr. This workflow avoids the separate overnight incubation step and uses Liberase TL to facilitate rapid tissue dissociation (Willenborg et al., 2022). Because Liberase TL provides strong enzymatic dissociation, the digestion time should be carefully controlled. Therefore, prepare all reagents, equipment, and collection tubes in advance before starting the protocol.

### Additional Materials (also see Basic Protocol)

1 mg/ml Liberase TL Research Grade (Sigma‐Aldrich, 5401020001)
2‐ml microcentrifuge (Eppendorf) tubes
Shaker incubator

#### Day 1: Obtaining skin biopsies

1 Follow the same procedure described in the Basic Protocol, steps 1‐3.

#### Day 1: Mechanical and enzymatic digestion of the skin (timing: ∼2 hr)

*NOTE*: These steps should be conducted at room temperature in a sterile environment when the cells are intended for sequencing. Perform the procedure under biosafety level 1 (BSL‐1) conditions.

2 Prepare skin digestion mix III (see recipe in Reagents and Solutions) by adding DNase I at 1/20, collagenase IV at 1/13, and Liberase TL at 1/33 of the prepared stock solutions to skin digestion medium. Keep the digestion mix on ice until use.
3 Transfer the skin piece to a 2‐ml microcentrifuge tube and finely cut up the skin directly in the tube using sterile surgical scissors. We usually cut the skin ∼150–180 times, depending on the sample size, with fewer cuts required for smaller pieces.
4 Add 1.5 ml of skin digestion mix III (from step 2) to the cut skin.
5 Close the tube and manually shake to mix well.
6 Incubate for 60 min at 37°C, 300 rpm, in a shaker incubator. Secure the microcentrifuge tube to the shaker using tape to prevent the tube from moving during incubation.
7 At the 30‐min time point, remove the tube from the shaker incubator and manually shake to mix the tissue before returning it to the incubator.
8 Once the incubation is complete, transfer the digested skin mixture through a 40‐µm‐pore‐size filter into a 50‐ml Falcon tube. Rinse the inside of the microcentrifuge tube with 1 ml FACS buffer and pass the rinse through the same filter.
9 Centrifuge for 5 min at 450 × *g*, 4°C.
10 Proceed to live/dead staining or conventional antibody staining and downstream flow cytometry as described in the Basic Protocol, steps 21‐35.

## REAGENTS AND SOLUTIONS

### FACS buffer

**Table:** 

Reagent	Final concentration	Amount
PBS	N/A	500 ml
BSA powder	0.5% (w/v)	2.5 g
0.5 M EDTA stock	2 mM	2 ml

The amounts listed are for one sample. Adjust the quantities as needed based on the number of samples.

### Skin digestion medium

**Table:** 

Reagent	Final concentration	Amount
RPMI‐1640 GlutaMAX	N/A	470 ml
10,000 U/ml penicillin‐streptomycin stock	100 U/ml	5 ml
1 M HEPES stock	32 mM	16 ml
Fetal bovine serum (FBS), heat inactivated	2%	10 ml

The amounts listed are for one sample. Adjust the quantities as needed based on the number of samples.

### Skin digestion mix I

**Table:** 

Reagent	Final concentration	Amount
Skin digestion medium (see recipe)	N/A	1.976 ml
100 mg/ml dispase II (Roche, 04942078001) stock	2 mg/ml	24 µl (1/50)

The amounts listed are for one sample. Adjust the quantities as needed based on the number of samples.

### Skin digestion mix II

**Table:** 

Reagent	Final concentration	Amount
Skin digestion medium (see recipe)	N/A	1.822 ml
10 mg/ml DNase I (Roche, 11284932001) stock	0.12 mg/ml	24 µl (1/20)
20 mg/ml collagenase IV (Sigma‐Aldrich, C4‐28‐100MG) stock	1.54 mg/ml	154 µl (1/13)

The amounts listed are for one sample. Adjust the quantities as needed based on the number of samples.

### Skin digestion mix III (fast digest)

**Table:** 

Reagent	Final concentration	Amount
Skin digestion medium (see recipe)	N/A	1.762 ml
10 mg/ml DNase I (Roche, 11284932001) stock	0.12 mg/ml	24 µl (1/20)
20 mg/ml collagenase IV (Sigma‐Aldrich, C4‐28‐100MG) stock	1.54 mg/ml	154 µl (1/13)
1 mg/ml Liberase TL Research Grade (Sigma‐Aldrich, 5401020001) stock	0.03 mg/ml	60 µl (1/33)

The amounts listed are for one sample. Adjust the quantities as needed based on the number of samples.

## COMMENTARY

### Critical Parameters

Maintain consistent skin biopsy size and processing time between samples. Ensure complete tissue immersion during dispase II incubation and cut the skin finely before enzymatic digestion. Follow digestion times and temperatures closely, particularly for the fast‐digest workflow, and minimize delays between tissue collection, digestion, and downstream processing.

### Troubleshooting

Table 2 lists problems that may arise with these procedures, along with their possible causes and solutions.

**Table 2:** Troubleshooting

Problem	Possible cause	Solution
Low cell yield	Incomplete tissue digestion	Finely mince the skin and ensure that each tissue piece is fully immersed in the digestion medium. Gently swirl to separate tissue fragments and ensure that the digestion medium reaches all surfaces. Avoid leaving tissue pieces in clumps.
Low cell viability	Over‐digestion or prolonged processing	Follow digestion times closely and avoid over‐digestion. Keep samples on ice when indicated.
Low recovery of epidermal immune cells, including Langerhans cells	Insufficient tissue dissociation during the long‐digest workflow	Ensure that the skin is fully immersed in the dispase II solution during the overnight incubation at 4°C before proceeding with the subsequent digestion steps.
Poor staining or loss of surface markers	Excessive enzymatic digestion	Avoid extending digestion times beyond those specified. Liberase TL provides strong proteolytic activity; therefore, strictly adhere to the specified digestion time to minimize potential loss or alteration of surface markers.
Low cell recovery after filtration	Cell loss during transfer or filtration	Rinse the tube and filter with FACS buffer after transferring the sample and before centrifugation to maximize cell recovery.

### Understanding Results

Figure 1 shows representative results from flow cytometry for the identification of immune cell populations in mouse skin wounds 2 days after wounding using the fast‐digest protocol.

**Figure 1:**
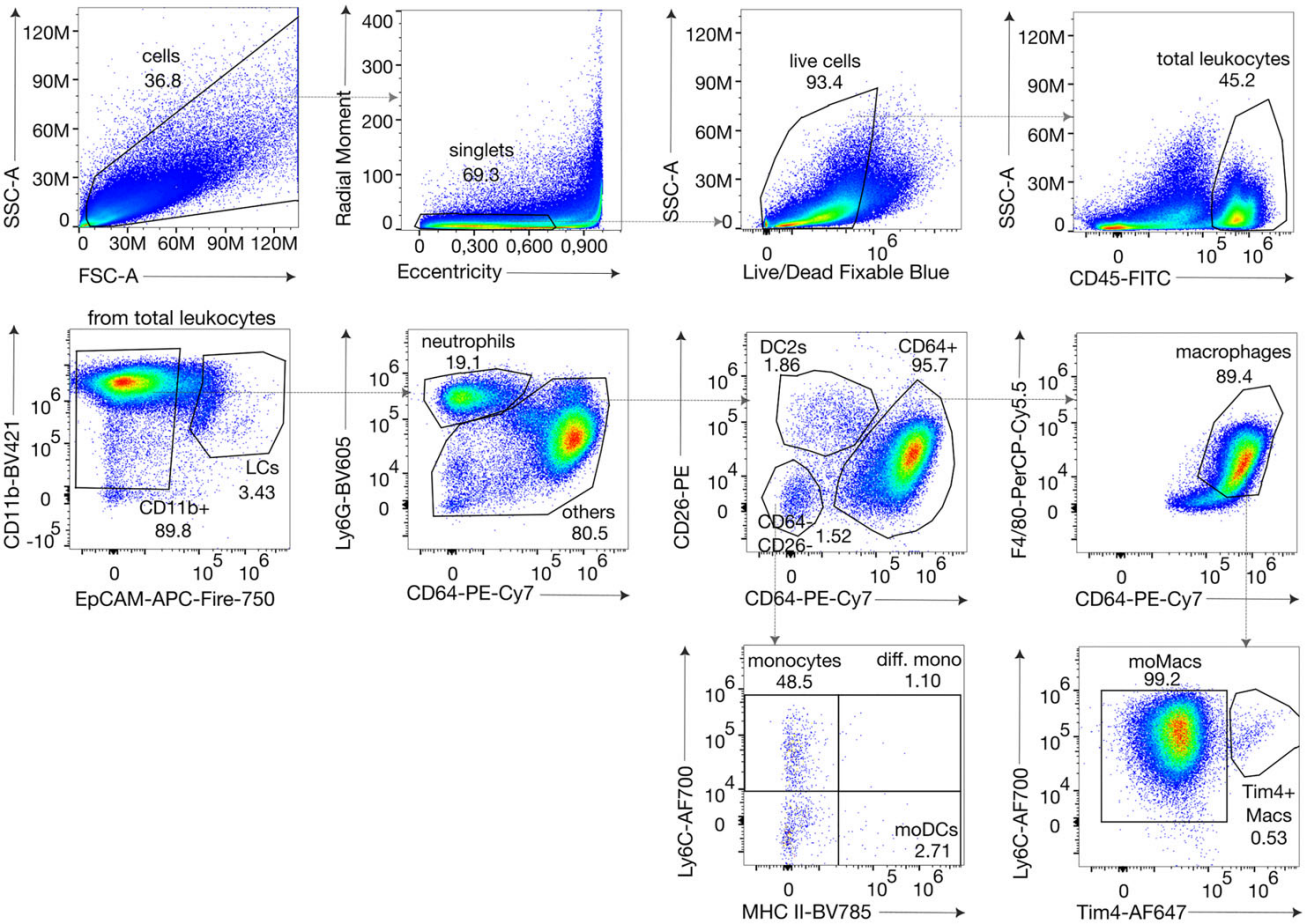
Representative flow cytometry gating strategy and results for the identification of immune cell populations in mouse skin wounds 2 days after wounding using the fast‐digest protocol. LCs, Langerhans cells; DC2s, type 2 conventional dendritic cells; diff. mono, differentiating monocytes; moDCs, monocyte‐derived dendritic cells; moMacs, monocyte‐derived macrophages; Macs, macrophages.

### Time Considerations

The basic workflow takes ∼2 days, including an overnight incubation. The fast‐digest workflow takes ∼2 hr and requires all reagents and equipment to be prepared in advance.

### Author Contributions

**Darawan Tabtim‐On**: Conceptualization; investigation; writing—original draft; methodology; formal analysis. **Mathieu Vetter**: Investigation; writing—review and editing. **Elodie Segura**: Conceptualization; methodology; writing—original draft; supervision; funding acquisition.

### Conflict of Interest

The authors declare no competing interest.

## Data Availability

Data sharing not applicable—no new data generated.
